# Site-Of-Care Viscoelastic Assay in Major Trauma Improves Outcomes and Is Cost Neutral Compared with Standard Coagulation Tests

**DOI:** 10.3390/diagnostics10070486

**Published:** 2020-07-17

**Authors:** Catriona Cochrane, Shalini Chinna, Ju Young Um, Joao D. Dias, Jan Hartmann, Jim Bradley, Adam Brooks

**Affiliations:** 1 Major Trauma, East Midlands Major Trauma Centre, Queen’s Medical Centre Nottingham, Nottingham NG7 2UH, UK; catriona.cochrane13@googlemail.com (C.C.); shalini.chinna@nhs.net (S.C.); umju.young@nhs.net (J.Y.U.); 2 Haemonetics Corporation, Boston, MA 02110, USA; jdias@haemonetics.com (J.D.D.); jan.hartmann@haemonetics.com (J.H.); 3 Department of Anaesthetics, Nottingham University Hospitals, Nottingham NG5 1PB, UK; Jim.Bradley@nuh.nhs.uk

**Keywords:** thromboelastography, TEG 6s, TEG, trauma, cost

## Abstract

Major hemorrhage is often associated with trauma-induced coagulopathy. Targeted blood product replacement could achieve faster hemostasis and reduce mortality. This study aimed to investigate whether thromboelastography (TEG^®^) goal-directed transfusion improved blood utilization, reduced mortality, and was cost effective. Data were prospectively collected in a U.K. level 1 trauma center, in patients with major hemorrhage one year pre- and post-implementation of TEG^®^ 6s Hemostasis Analyzers. Mortality, units of blood products transfused, and costs were compared between groups. Patient demographics in pre-TEG (*n* = 126) and post-TEG (*n* = 175) groups were similar. Mortality was significantly lower in the post-TEG group at 24 h (13% vs. 5%; *p* = 0.006) and at 30 days (25% vs. 11%; *p* = 0.002), with no difference in the number or ratio of blood products transfused. Cost of blood products transfused was comparable, with the exception of platelets (average £38 higher post-TEG). Blood product wastage was significantly lower in the post-TEG group (1.8 ± 2.1 vs. 1.1 ± 2.0; *p* = 0.002). No statistically significant difference in cost was observed between the two groups (£753 ± 651 pre-TEG; £830 ± 847 post-TEG; *p* = 0.41). These results demonstrate TEG 6s-driven resuscitation algorithms are associated with reduced mortality, reduced blood product wastage, and are cost neutral compared to standard coagulation tests.

## 1. Introduction

Thirty percent of patients with major hemorrhage in trauma develop associated trauma-induced coagulopathy (TIC) [1,2,3]. The wars in Iraq and Afghanistan focused attention on trauma resuscitation and the use of blood and blood products in a 1:1:1 ratio. Subsequently, the Pragmatic Randomized Optimal Platelet and Plasma Ratios (PROPPR) and Prospective, Observational, Multicenter, Major Trauma Transfusion (PROMMTT) trials [4,5] aimed to define both the value of the 1:1:1 transfusion ratio on outcomes and whether a 1:1:2 ratio was equivalent or better.

Following current practices, a trauma patient presenting with major hemorrhage will activate the massive transfusion protocol (MTP) and is categorized as a code red patient. This results in them receiving multiple blood products such as packed red blood cells (RBCs), platelets, and fresh frozen plasma (FFP) [6,7,8]. Further blood product requirement is often directed by routine coagulation testing, such as PT (prothrombin time) and APTT (activated partial thromboplastin time). However, the use of these tests in such a dynamic situation can end up providing historical results due to the time required for results to become available, impairing decision-making. Site-of-care viscoelastic testing, as recommended by the European and U.S. trauma guidelines, [7,9,10] may be beneficial in the trauma setting to guide blood product usage.

Thromboelastography (TEG^®^ analyzer) is a viscoelastic method that shows a dynamic clotting profile. With the development of the TEG^®^ 6s Hemostasis Analyzer (Haemonetics Corp., Boston, MA, USA), testing is available at the site of care providing rapid, individual patient coagulation assessment. Viscoelastic assays are thought to be more reliable than conventional clotting tests, providing a more accurate and rapid assessment of coagulopathy that is more predictive of massive transfusion needs [3,11,12,13,14,15,16,17,18,19,20].

Managing uncontrollable hemorrhage of the trauma patient more effectively with target-driven blood product replacement could achieve hemostasis quicker and, therefore, reduce mortality due to hemorrhage in trauma [21]. Following a predetermined algorithm and goal-directing transfusions to individual patients could make blood product utilization more efficient and, therefore, reduce the overall cost of transfusions [7].

The use of TEG has shown promise in the goal-directed use of blood products in the resuscitation of the hemorrhaging major trauma patient [22,23,24]. Studies show that the TEG device provides an indication of which blood product the patient is deficient in, allowing a more goal-directed resuscitation transfusion algorithm. This allows the trauma team to replace specific blood products to counteract the deficiency, ultimately leading to potentially improved hemorrhage control, reduced costs, and more effective TIC control [22,23,24]. The TEG 6s device uses resonance-frequency viscoelasticity measurements with assays carried out using a multi-channel microfluidic cartridge and is associated with higher precision and greater ease of use compared with the TEG^®^ 5000 [25]. TEG 6s has also shown close correlation to the TEG 5000 device in clinical practice for the treatment of trauma patients, with improved within-device reliability [26].

The aim of this study was to investigate the effect of TEG 6s on mortality when used for goal-directed hemostatic therapy for massive hemorrhage in major trauma patients in a level one U.K. trauma center. The study also sought to establish whether TEG goal-directed MTP improved blood utilization during massive hemorrhage in major trauma and, subsequently, reduced the overall cost of transfusion.

## 2. Materials and Methods

### 2.1. Data Collection

Data were prospectively collected in a U.K. level 1 trauma center in patients who activated the major hemorrhage protocol one year pre- and one year post-implementation of a TEG 6s-driven transfusion algorithm into the center’s code red resuscitation protocol. Inclusion criteria were major hemorrhage protocol activation, code red trauma call, code amber trauma call with suspicion of significant active bleeding, and blood transfusion commenced in a trauma patient. We excluded patients who were secondary transfers from other trauma units, major trauma patients who activated the MTP 24 h post admission, age < 16, pregnant patients, and non-trauma patients activating the major hemorrhage protocol.

Data were collected on age, gender, mechanism, site of injury, injury severity score (ISS), shock index (SI), blood products used and wasted, outcomes at 24 h, and TEG results. Cost was defined as overall blood product usage and TEG cartridge cost within initial 24 h and wastage of blood products requested but not transfused and not re-issued by blood bank.

### 2.2. Thromboelastography 

**Figure 1:**
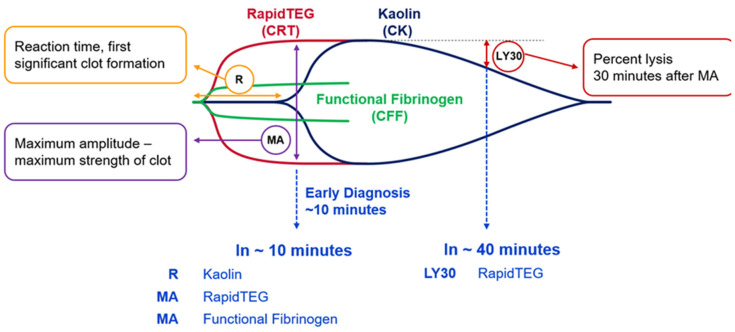
Thromboelastography (TEG) measurements shown in graphical format explaining the results of the relevant areas of the curve. LY30: Hyperfibrinolysis after 30 min suggests the stability of the clot formed; MA: maximum amplitude is suggestive of the strength of the clot formed; R: reaction time and length of time taken to initiate clot formation.

The TEG 6s Hemostasis Analyzer (Haemonetics Corp., Boston, MA, USA) is a new-generation thromboelastography device used at the site of care, which requires ~0.6 mL of titrated blood taken from a 2 mL sodium citrate vacutainer tube. The full results of the TEG 6s sample are available in just over 30 min, but critical clinically relevant results and information are available within 2 min (Figure 1). An initial TEG 6s sample and coagulation sample were taken immediately on admission of trauma patients who fitted the inclusion criteria, and, if clinically indicated, transfusion was commenced with pack 1 of our MTP as described below. We then continued to use TEG 6s to guide the patients’ blood transfusion by taking a TEG 6s sample for every 4 units of red cells transfused, 6 h after hemostasis had occurred (defined as surgical hemostasis or when patient no longer required blood product transfusion, e.g., fresh frozen plasma (FFP), Octaplas, platelets, or cryoprecipitate), and 24 h after admission to assess the sustainability of clot and evidence of missed coagulopathy.

### 2.3. Massive Transfusion Protocol (MTP)

The MTP comprised the following: Pack 1: 4 units of packed RBCs and 2 units of FFP; Pack 2: 6 units of RBCs, 5 units of Octaplas, 1 adult unit of platelets, 2 units of cryoprecipitate; Pack 3: the same as pack 2. As per the Clinical Randomization of an Antifibrinolytic in Significant Hemorrhage 2 (CRASH-2) study [27], tranexamic acid was given to all patients prior to their arrival at the trauma center.

### 2.4. Pre-TEG Group

In the retrospective control study group, the patient was assessed by the trauma team leader as requiring activation of the MTP and this was continued or stood down as the clinical picture necessitated and evolved. Standard coagulation tests were taken and used to direct MTP if still clinically relevant. Coagulation test reference ranges in our hospital trust were as follows: PT: 10.0–12.0, APTT: 21.0–29.0, thrombin time (TT): 13.0–20.0, fibrinogen: 1.80–4.00. The MTP policy was to use the following values to goal-direct blood transfusion when using standard coagulation assays to maintain the following standard coagulation results: platelets > 75 × 109/L, PT/APTT < 1.5 × normal, fibrinogen > 1.5–2.0 g/L.

### 2.5. Post-TEG Group

**Figure 2:**
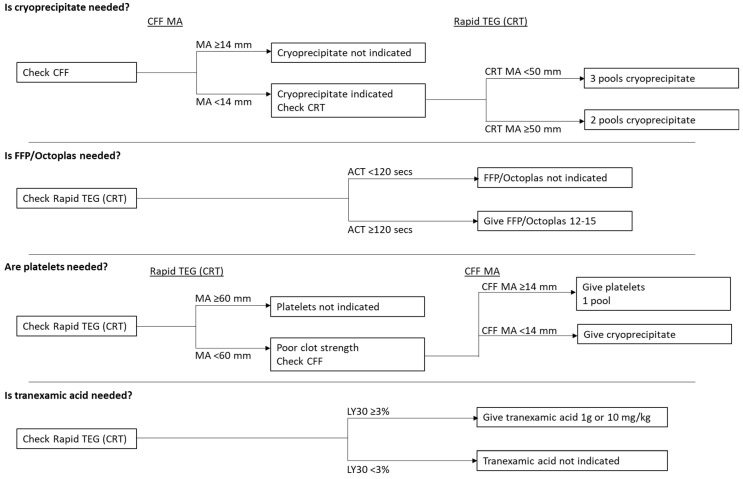
Major Trauma TEG 6s protocol. ACT, TEG activated clotting time; CFF, citrated functional fibrinogen; CRT, citrated RapidTEG; FFP, fresh frozen plasma; LY30, lysis 30; MA, maximal amplitude.

In the post-TEG group, pack 1 of the MTP was given as a standard course and then the TEG 6s sample was used to guide transfusion of product alongside the transfusion of RBCs (Figure 2). The reference ranges for a normal RapidTEG assay are as follows: activated clotting time (ACT) 82–152 s, maximal amplitude (MA) 52–70 mm, and lysis 30 (LY30) 0.0–2.2%. The reference range for a normal functional fibrinogen assay is MA 15–30 mm. The major trauma algorithm was based on an earlier version of the published implementing Treatment Algorithms for the Correction of Trauma-Induced Coagulopathy (iTACTIC) trial algorithm [28], which underwent several subsequent revisions. While this algorithm was initially generated for the TEG 5000, validation data showed equivalent values were applicable for the TEG 6s, which was used in the iTACTIC trial [25,26]. Data from the validation and method comparison studies by Gurbel et al. [25] and Neal et al. [26] demonstrate that the TEG 6s device has close correlation to the TEG 5000 device with improved inter-device reliability. At the time the iTACTIC trial algorithm changed to its final published form, the study had already started using the earlier version of the algorithm for clinical decision-making. Retrospective calculations using the newer citrated functional fibrinogen (CFF)-MA target indicated that it would have caused many more units of cryoprecipitate to be administered with no apparent benefit, and therefore, the study continued with the original thresholds shown in Figure 2.

Initial RapidTEG ACT < 120 s with no clinical evidence of bleeding was used to stand down the MTP prior to transfusion. If TEG 6s was not available, the MTP was given as standard.

If there was suspicion of hemorrhage, but the patient did not require immediate transfusion of blood product, a TEG 6s sample was taken to assess their coagulopathic state and the result used to guide the clinician as to whether or not to activate the MTP. Despite the initial TEG 6 s sample occasionally being normal or near normal on a hemodynamically unstable patient, we encouraged the use of the leading clinician’s clinical decision-making in order to hold or continue blood transfusion and to reassess coagulopathy status after 4 units of RBCs. We collected blood transfusion data for the first 24 h period after the patient was admitted.

### 2.6. Economic Analysis

The cost of blood products transfused was compared between groups, for individual products and in total. The costs of TEG cartridges and standard coagulation tests were also considered. The cost of hospital length of stay was not included as this is likely to be affected by multiple factors. For the subgroup of patients with wastage data, the cost of wasted blood products was also factored into the analysis. As with blood product usage, data on the cost of transfused units were available for all patients. However, costs of wasted products were not available for all patients, and hence fewer patients were included in the analyses involving this information.

### 2.7. Statistical Analysis

All analyses compared the pre- and post-TEG groups. A pragmatic time period of one year pre- and post-implementation of TEG 6s was chosen to inform the number of patients in the study. The groups were compared in terms of demographics, injury characteristics, and baseline variables. Continuous variables were compared between groups using the unpaired *t*-test, if found to be normally distributed, and the Mann–Whitney test if not. The chi-square test was used to compare categorical variables between groups.

The units of blood products transfused were also compared between groups. All blood product outcomes were found to have a positively skewed distribution, and so the Mann–Whitney test was used for these analyses. Equivalent statistical methods were used to compare the number of units wasted between groups. The chi-square test was used to compare the occurrence of any transfusion, and a massive transfusion, between the pre- and post-TEG groups.

The costs of blood products transfused and wasted was compared between groups. The number of units of blood products and the cost outcomes were found to follow positively skewed distributions. Therefore, a bootstrapping approach was used to compare the costs of the two groups. This involved resampling data at random from each cohort multiple times, to generate a confidence interval for the mean cost difference between groups. The cost of TEG cartridges was factored in to create the total cost for each patient.

Other outcomes compared between groups in the final analysis included: total hospital length of stay (LOS), compared between groups using the Mann–Whitney test as it was found to have a positively skewed distribution; and mortality at 24 h and 30 days, which was compared using a chi-squared test.

## 3. Results

### 3.1. Patient Demographics

There were 126 patients in the pre-TEG group and 175 in the post-TEG group. There was no statistical difference between the groups for ISS, SI, injury site, and mechanism, excluding gender (the post-TEG group had fewer females, *p* < 0.05). There was an average of 1.8 cartridges per post-TEG patient with the majority of patients (61%) requiring just 1 cartridge (Table 1).

### 3.2. Mortality

Mortality at both 24 h and 30 days was significantly lower in the post-TEG group compared to the pre-TEG group. Only 5% (*n* = 8/175) of patients in the post-TEG group died within 24 h, compared to 13% (*n* = 17/126) in the pre-TEG group, a difference that was statistically significant (*p* = 0.006). Mortality at day 30 in the pre-TEG group was more than double that of the post-TEG group (25% [*n* = 32/126] compared to 11% [*n* = 20/175]; *p* = 0.002). However, total hospital LOS was significantly greater in the post-TEG group (*p* < 0.001), with a median LOS of 14 days, compared to a median of 9 days for the pre-TEG group in line with survival bias (Table 2).

### 3.3. Blood Product Usage

There was no difference between groups in the number of blood products transfused, although there was a trend for increased use of platelets in the post-TEG group (0.2 ± 0.5 pre-TEG; 0.4 ± 0.8 post-TEG; *p* = 0.05). The occurrence of any transfusion was slightly lower in the post-TEG group (86% pre-TEG vs. 78% post-TEG; *p* = 0.08), although this difference did not quite reach statistical significance. Conversely, the occurrence of a massive transfusion was slightly higher in the post-TEG group (3% pre-TEG; 8% post-TEG; *p* = 0.08), but this difference did not reach statistical significance (Table 3). The ratio of blood:FFP:platelets transfused was similar between groups (1.00:0.70:0.24 pre-TEG; 1.00:0.67:0.42 post-TEG).

The results for blood products wasted suggested that FFP/Octaplas (1.6 ± 2.0 pre-TEG; 0.9 ± 1.8 post-TEG; <0.01) and RBC (0.2 ± 0.7 pre-TEG; 0.1 ± 0.5 post-TEG; *p* < 0.02) wastage was significantly lower in the post-TEG group. Additionally, the overall blood product wastage was significantly lower in the post-TEG group (1.8 ± 2.1 pre-TEG; 1.1 ± 2.0 post-TEG; *p* = 0.002). A mean of 1.1 units was wasted per patient in the post-TEG group, compared to 1.8 units in the pre-TEG group (Table 3). More post-TEG patients had no transfusion, as the MTP was stood down prior to transfusion.

### 3.4. Cost Outcomes

The cost of blood products transfused was comparable between groups with the exception of platelets. Results suggest that there was a significantly higher cost of platelets in the post-TEG group than in the pre-TEG group (41 ± 90 pre-TEG; 79 ± 152 post-TEG; *p* = 0.008), on average by £38. However, the overall cost of transfusions did not significantly vary between the groups (625 ± 655 pre-TEG; 678 ± 786 post-TEG; *p* = 0.52). When the total cost, including the cost of TEG cartridges, was taken into account, patients in the post-TEG group had a mean cost of £127 per patient more than the pre-TEG patients (625 ± 655 pre-TEG; 753 ± 828 post-TEG; *p* = 0.14); however, this difference did not reach statistical significance (Table 4). Wastage of blood products overall was significantly lower in the post-TEG group (127 ± 146 pre-TEG; 74 ± 133 post-TEG; *p* = 0.002), by a mean of £53. (Table 3). In particular, wastage of FFP/Octaplas was significantly higher in the pre-TEG group (99 ± 128 pre-TEG; 57 ± 112; *p* = 0.004), by a mean of £42 per patient.

When the data on blood product utilization, wastage, and the costs of TEG cartridges is taken into account the results suggest no evidence of a statistically significant difference between the overall cost in the two groups over the initial 24 h (753 ± 651 pre-TEG; 830 ± 847 post-TEG; *p* = 0.41; Table 4).

## 4. Discussion

This study demonstrates that the use of TEG 6s in a trauma setting improves patient outcomes and is also cost neutral compared with standard coagulation tests. In this prospective study of 301 patients treated at a U.K. level 1 trauma center, TEG 6s was associated with significantly improved mortality at 24 h and at 30 days, although average hospital LOS was greater in the post-TEG group, most likely due to survival bias. The use of blood products between the groups was comparable and although there was a trend for increased use of platelets in the post-TEG group, wastage of blood products was significantly lower following use of TEG 6s. Consequently, economic analysis of the use of TEG 6s demonstrates that it is cost neutral when compared to the pre-TEG group.

TIC can exacerbate severe bleeding in trauma patients and there is a need for rapid site-of-care management to guide transfusion and improve outcomes. The time needed to turnaround standard coagulation tests varies; however, it is generally accepted that the time taken is too long (typically 45–60 min to run the tests not including time taken to transfer the samples and results to/from the hospital laboratory) [29]. Viscoelastic monitoring using thromboelastography (TEG) is well positioned to quickly assess a patient’s coagulation profile—initial results can be obtained in as little as 10 min with full results available after 30–60 min directly at the site of care [29,30,31,32]. TEG is also able to guide hemostatic replacement according to individual patient needs [21] and has an advantage over conventional coagulation tests in the treatment of trauma [20]. As a result, use of TEG has been associated with improved patient outcomes, particularly reduction in mortality as demonstrated in this study. Reductions in mortality have also been demonstrated in previous studies; a recent randomized clinical trial demonstrated improved mortality at 28 days following TEG compared to standard coagulation tests (19.6% TEG; 36.4% standard coagulation tests; *p* = 0.049) [21]. A further study also demonstrated that TEG-defined hypercoagulable patients had lower 24 h mortality (0.0% vs. 5.5% vs. 27.8%, adjusted *p* < 0.001) and lower 7 day mortality (0.0% vs. 5.5% vs. 36.1%, adjusted *p* < 0.001) compared to those with a normal TEG profile or hypocoagulable patients [33]. Several observational studies have also examined the effect of TEG-guided transfusion on mortality outcomes; whilst some small studies demonstrated lower mortality [3,34,35,36] others demonstrated no overall difference in mortality outcomes [34,37,38]. Use of TEG or rotational thromboelastometry (ROTEM) has also been shown to be predictive of coagulopathy-related death in several studies [34]. The TEG 6s device has been validated against the TEG 5000 [25] and has shown high reliability and efficacy for use in trauma treatment [26]. The TEG 6s device is also easier to use due to the cartridge system and less sensitive to surrounding vibrations and temperature compared with the TEG 5000 [37]. This makes the device highly suitable for use in trauma patients at the site of care, which may not be located in a traditional hospital setting.

This study did not demonstrate a difference in the overall number of blood products transfused between the pre- and post-TEG groups; however, reduction in blood product usage following TEG has been demonstrated. A cohort study demonstrated lower exposure to blood products with ROTEM-guided transfusion—red blood cell transfusion was avoided in 29% of ROTEM-guided patients, compared to 3% in the routine coagulation test-guided groups (*p* < 0.001) [37]. Similarly, a modeling study suggested that rapid-TEG guided transfusion would reduce the proportion of patients requiring blood products from 73.1% to 53.9% (*p* = 0.03) [39]. Interestingly, in this study, there was a slightly higher occurrence of massive transfusion in the post-TEG group. This may be due to the increased sensitivity but reduced specificity of viscoelastic tests to identify or predict the need for massive transfusion [31,40,41]. There was also a significant increase in platelet transfusion in the post-TEG group in this study—this may be due to the ability of the viscoelastic test to better identify trauma-induced coagulopathies [34]. In particular, viscoelastic tests have demonstrated ability to detect platelet dysfunction in trauma patients and have shown lower platelet response [42], increased platelet dysfunction [43,44], and lower platelet component of clot elasticity in trauma patients [45].

Importantly, this study aimed to assess the cost effectiveness of TEG 6s in trauma care. There is little previous literature surrounding the cost effectiveness of viscoelastic monitoring and guidance in the trauma setting. A previous economic evaluation was performed for NICE (National Institute for Health and Care Excellence). Within this evaluation, both TEG and ROTEM were found to be more cost effective than standard care tests, with the per patient saving estimated as £688 for ROTEM and £721 for TEG [46]. However, as there were limited data available from trauma patients, results were extrapolated from other populations. This study therefore addresses a major need in the trauma care setting. Consistent with the findings of Whiting et al. 2015 [46], these data suggest that use of TEG 6s is cost neutral compared to standard coagulation tests when used to treat trauma patients. Although the overall costs of transfusions (including the cost of the TEG cartridge) was increased in the post-TEG group, there was a reduction in wastage of all blood products in this group compared to the pre-TEG group. This is of particular importance given that blood product resource is highly valuable, and its collection, processing, and storage are associated with significant costs.

The results from this study are highly supportive of the use of TEG 6s for the treatment of trauma patients. However, there are a few limitations that should be taken into account. This was a prospective study, and as such, patients were not randomized to TEG/standard monitoring and a control group was not utilized. Instead, the group was compared to a retrospective control—namely patients who were treated one year prior to the introduction of TEG-guided therapy. The results from this study also only represent the findings at one single center within the U.K., further multi-center and larger studies will be needed to fully assess the efficacy and cost-effectiveness of viscoelastic monitoring for the treatment of trauma patients.

## 5. Conclusions

In conclusion, the introduction of site-of-care viscoelastic testing is becoming more commonplace for the treatment of trauma and major bleeding. These results demonstrate that TEG 6s-driven resuscitation algorithms are associated with comparable blood product utilization and reduced blood product wastage and are therefore cost neutral when compared to standard coagulation tests. Use of TEG 6s also demonstrated improvement in mortality independent of the transfusion ratio, although the mechanism of this needs further assessment.

## Figures and Tables

**Table 1:** Summary of demographics and baseline characteristics.

Outcome	Category	Pre-TEG (*n* = 126)	Post-TEG (*n* = 175)	*p*-Value
Age	-	47.2 ± 23.6	43.1 ± 20.2	0.11
Sex	Male	84 (67%)	137 (78%)	**0.02**
	Female	42 (33%)	38 (22%)	
Shock index	-	0.87 [0.69, 1.08]	0.82 [0.65, 1.10]	0.78
GCS	-	15 [8, 15]	14 [8, 15]	0.37
Injury severity score	-	25 [13, 33]	22 [13, 34]	0.75
Mechanism of injury	Blunt	107 (85%)	149 (85%)	0.96
	Penetrating	19 (15%)	26 (15%)	
Injury site ^1^	Head	39 (35%)	54 (31%)	0.54
	Chest	61 (54%)	95 (55%)	0.92
	Abdominal	42 (37%)	71 (41%)	0.54
	Pelvis	28 (25%)	33 (19%)	0.24
	MSK	65 (58%)	103 (59%)	0.78
Lactate	-	3.4 [2.4, 5.6]	3.0 [1.9, 4.5]	0.06
TEG cartridges	-	-	1.8 ± 1.4	-
TEG cartridges	1	-	107 (61%)	-
(categorical)	2	-	35 (20%)	
	3	-	17 (10%)	
	4+	-	16 (9%)	

GCS, Glasgow Coma Scale; MSK, musculoskeletal. Summary statistics are: Mean ± standard deviation, Median [inter-quartile range], or Number (percentage). *p* values < 0.05 are in bold to indicate significance. ^1^ Patients could have more than one injury site.

**Table 2:** Mortality.

Outcome	Pre-TEG (*n* = 126)	Post-TEG (*n* = 175)	*p*-Value
Total hospital LOS	9 [3, 19]	14 [6, 27]	**<0.001**
24 h mortality	17 (13%)	8 (5%)	**0.006**
30 day mortality	32 (25%)	20 (11%)	**0.002**

Summary statistics are: Median [inter-quartile range], or Number (percentage). LOS, length of stay. *p* values < 0.05 are in bold to indicate significance.

**Table 3:** Blood product usage.

Outcome	Pre-TEG (*n* = 126)	Post-TEG (*n* = 175)	*p*-Value
**Units transfused**			
RBC	3.6 ± 3.3	3.9 ± 4.0	0.91
FFP/Octaplas	2.5 ± 3.4	2.6 ± 3.8	0.98
Platelets	0.2 ± 0.5	0.4 ± 0.8	0.05
Cryoprecipitate	0.3 ± 1.0	0.5 ± 1.1	0.14
All products combined	6.7 ± 7.5	7.5 ± 8.8	0.94
Any transfusion	108 (86%)	136 (78%)	0.08
Massive transfusion ^1^	4 (3%)	14 (8%)	0.08
Massive transfusion ^2^	27 (21%)	41 (23%)	0.68
**Outcome**	**Pre-TEG** **(*n* = 126)**	**Post-TEG** **(*n* = 129)**	***p*-Value**
**Units wasted**			
RBC	0.2 ± 0.7	0.1 ± 0.5	**0.02**
FFP/Octaplas	1.6 ± 2.0	0.9 ± 1.8	**0.004**
Platelets	0.0 ± 0.1	0.0 ± 0.1	0.58
Cryoprecipitate	0.0 ± 0.3	0.1 ± 0.5	0.10
All products combined	1.8 ± 2.1	1.1 ± 2.0	**0.002**

Summary statistics are as follows: Mean ± standard deviation, or Number (percentage). ^1^ Defined as >10 units of RBCs transfused; ^2^ defined as >10 units of any blood product transfused. *p* values < 0.05 are in bold to indicate significance.

**Table 4:** Cost outcomes.

Outcome	Pre-TEG Mean ± SD (*n* = 126)	Post-TEG Mean ± SD (*n* = 175)	Difference ^1^ Mean (95% CI)	*p*-Value
**Units transfused**				
RBC	432 ± 401	472 ± 480	40 (−54, 133)	0.42
FFP/Octaplas	142 ± 196	111 ± 191	−30 (−67, 17)	0.18
Platelets	41 ± 90	79 ± 152	38 (10, 68)	**0.008**
Cryoprecipitate	10 ± 32	15 ± 36	5 (−3, 11)	0.24
All products combined	625 ± 655	678 ± 786	53 (−91, 227)	0.52
TEG cartridges	-	74 ± 59		-
Total cost 1 ^2^	625 ± 655	753 ± 828	127 (−22, 308)	0.14
**Units wasted**				
RBC	26 ± 80	10 ± 60	−15 (−33, 1)	0.07
FFP/Octaplas	99 ± 128	57 ± 112	−42 (−69, −14)	**0.004**
Platelets	2 ± 17	3 ± 24	1 (−3, 7)	0.57
Cryoprecipitate	1 ± 8	3 ± 14	2 (0, 5)	0.09
All products combined	127 ± 146	74 ± 133	−53 (−91, −17)	**0.002**
Total cost 2 ^3^	753 ± 651	830 ± 847	78 (−88, 304)	0.41

^1^ Difference calculated as post-TEG minus pre-TEG; ^2^ Cost calculated as cost of total units transfused plus TEG cartridges; ^3^ Cost calculated as cost of total units transfused, total units wasted plus TEG cartridges. *p* values < 0.05 are in bold to indicate significance
